# Endomyocardial Fibrosis Found Incidentally on Cardiac Imaging

**DOI:** 10.7759/cureus.17186

**Published:** 2021-08-15

**Authors:** Yesha Rana, Ramyashree Tummala, Bette Kim, Deepika Misra

**Affiliations:** 1 Department of Internal Medicine, Icahn School of Medicine at Mount Sinai, Mount Sinai Beth Israel Medical Center, New York City, USA; 2 Department of Cardiology, Icahn School of Medicine at Mount Sinai, Mount Sinai Beth Israel Medical Center, New York City, USA; 3 Department of Cardiology, Icahn School of Medicine at Mount Sinai, Mount Sinai West Hospital, New York City, USA

**Keywords:** endomyocardial fibrosis, echocardiography, thrombus, cardiac mri, cardiac imaging

## Abstract

Endomyocardial fibrosis (EMF) is a rare disease in the developed world characterized by the fibrosis of the endocardium in one or both of the ventricles causing restrictive-type cardiomyopathy. We present a case of a 47-year-old Chinese female with a past medical history of breast cancer treated in 2014 currently on tamoxifen therapy presented to the cardiology office for multiple presyncopal and syncopal events at rest. She was found to have apical hypertrophic cardiomyopathy (HCM) on echocardiogram. Subsequently, cardiac magnetic resonance imaging (cMRI) showed severe apical hypertrophy without left ventricular aneurysm, and evidence of small apical thrombus with subendocardial enhancement. There was no resolution of the left ventricular thrombus after a year-long course of therapeutic anticoagulation, a finding more consistent with EMF.* *Though the diagnosis of EMF initially depends on echocardiographic findings, cMRI is an essential imaging modality that allows clinicians to easily differentiate between potential diagnoses with the information that it provides. Early diagnosis, differentiation, and treatment for HCM are important for a good prognosis.

## Introduction

Endomyocardial fibrosis (EMF) is a rare disease in the developed world characterized by the fibrosis of the endocardium in one or both of the ventricles causing restrictive-type cardiomyopathy. Myocardial fibrosis usually occurs due to the deposition of collagen and fibroblast proliferation. Presentation of EMF can vary depending on the stage of EMF and extent of fibrosis, and it is known to be extremely rare in the developed world [1]. We present a case of EMF in a patient presenting with syncope, highlighting the role of cardiac imaging in diagnosis.

## Case presentation

A 47-year-old Chinese female with a past medical history of breast cancer treated in 2014 with lumpectomy and radiation, currently on tamoxifen therapy presented to the cardiology office for multiple presyncopal and syncopal events at rest. She described these events as sudden onset lasting only a few minutes with no prodrome. She had a previous episode in 2012 where she was found to be hypotensive; however, stress transthoracic echocardiogram (TTE) and Holter monitor were both negative for arrhythmias or structural changes. On further questioning, she denied having chest pain, shortness of breath, exercise intolerance, dizziness, or orthopnea. No significant family history for hypertrophic cardiomyopathy (HCM) or sudden cardiac death (SCD). Social history was noncontributory. Initial vital signs were stable, and the physical exam was unremarkable for any clinical signs of congestive heart failure, orthostasis, or murmurs.

**Figure 1:**
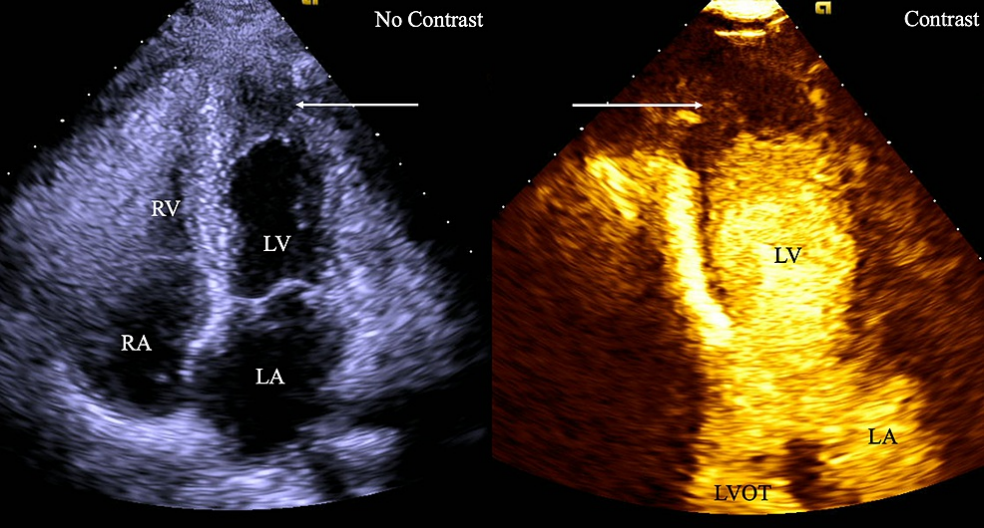
Apical four-chamber view (left) and apical three-chamber view with definity contrast (right) on echocardiography depicting apical hypertrophy and LV thrombus. RV - right ventricle, RA - right atrium, LV - left ventricle, LA - left atrium, LVOT - left ventricular outflow tract

Lab work showed no evidence of eosinophilia. Electrocardiogram at the initial visit was significant for normal sinus rhythm at 73 beats per minute with left ventricular hypertrophy and strain with diffuse T-wave inversions in leads V3-V6, right axis deviation, and Qtc 484. TTE and stress TTE demonstrated left ventricular ejection fraction (LVEF) of 65% without evidence of left ventricular outflow tract (LVOT) obstruction, ischemia, or exercise intolerance and apical HCM confirmed with Definity contrast (Figure 1). Subsequently, a cardiac magnetic resonance imaging (cMRI) was obtained and was significant for severe apical hypertrophy with thickness up to 1.5 cm without evidence of dynamic LVOT obstruction at rest, mild intramyocardial scarring consistent with HCM, and a small apical thrombus and subendocardial enhancement in the left ventricular apex without an aneurysm.

**Figure 2:**
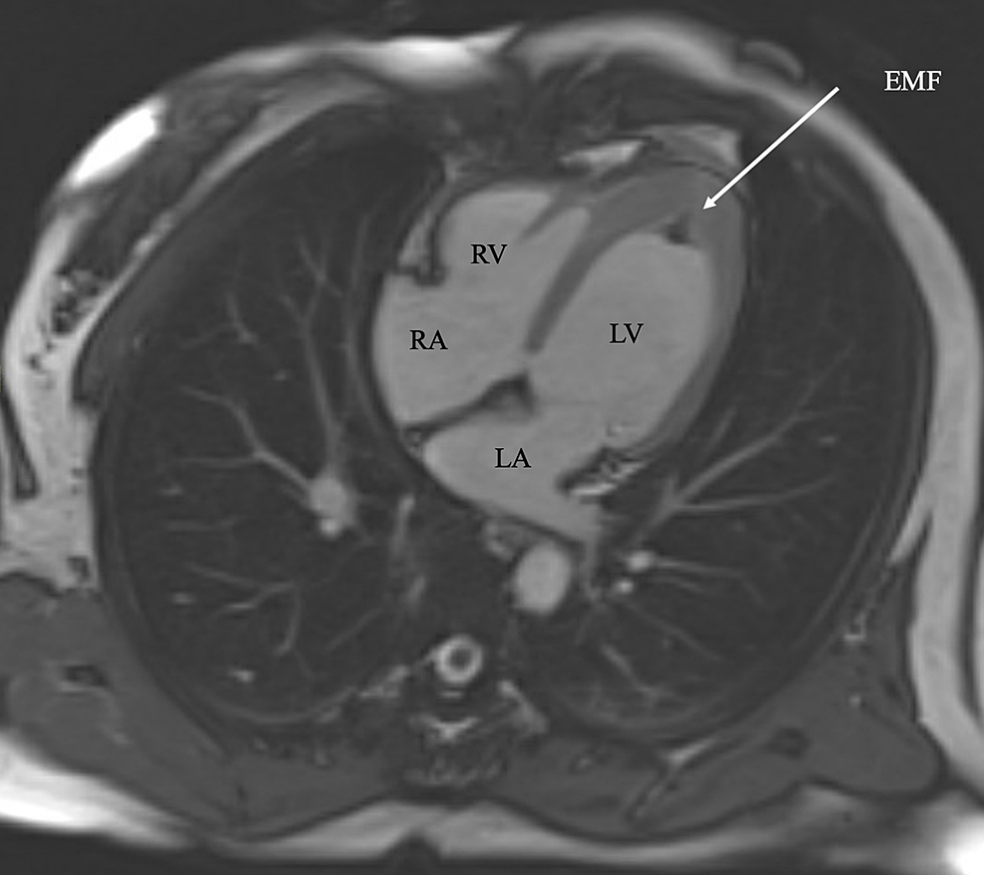
cMRI four-chamber view depicting endomyocardial fibrosis after the completion of a year-long course of anticoagulation with apixaban for LV thrombus. cMRI - cardiac magnetic resonance imaging, EMF - Endomyocardial fibrosis

She was started on a year-long course of anticoagulation with apixaban for the treatment of left ventricular thrombus as well as a beta-blocker for cardiomyopathy. Repeat cMRI after completion of the anticoagulation course showed a small persistent left ventricular thrombus, a finding that is more consistent with EMF rather than apical HCM (Figure 2).

## Discussion

EMF is a rare disease in North America and is typically common in the tropical and subtropical regions of the developing world [1,2]. In the United States, however, EMF has been found to be extremely rare and can be missed by unsuspecting eyes. Our patient who we present in this case is neither from a tropical region nor from an underserved background making it a very rare finding. This disease is mainly characterized by a gross change in the makeup of the endocardium of one or both of the ventricles. This change is particularly progressive and can ultimately lead to constriction of the affected ventricular cavities leading to restrictive cardiomyopathy.

Several etiologies have been implicated in the development of EMF including infectious sources, autoimmune, allergy mediated, and genetics [3]. It has also been suggested that factors, such as poverty, diet, environmental, and infections, may combine in an individual to give rise to an inflammatory process that leads to endomyocardial damage leading to scar formation [2]. Furthermore, several studies exploring the immune response in EMF have shown eosinophilia in patients with EMF. In our patient, lack of eosinophilia was used as a differentiating factor between hypereosinophilic syndrome and EMF [4].

Furthermore, our patient’s history of tamoxifen therapy and prior radiation exposure for breast cancer treatment does present a possible etiology for her fibrosis. Estrogen receptor modulators such as tamoxifen have been associated with higher rates of venous thromboembolic disease and stroke but have not been associated with fibrosis. A study performed by Lexow et al. in 2013 reported cardiac fibrosis in the mice model occurring in the acute phase after tamoxifen treatment and was attributed due to increased expression of pro-inflammatory cytokines [5]. Radiation therapy has been reported to contribute to the development of radiation-induced myocardial fibrosis (RIMF). Unlike EMF, RIMF is characterized by nonspecific, diffuse interstitial fibrosis that involves all layers of the heart [5]. In addition, it can present in three stages, acute phase occurring approximately six hours after radiation, latent phase occurring approximately two days after exposure, and late phase occurring 70 days after radiation [6]. The timing of our patient’s presentation paired with our imaging findings being limited to the endocardium support that these may be isolated events.

The natural history and presentation of EMF have been well documented as a progression from an active phase with recurrent active inflammatory processes evolving into restrictive-type cardiomyopathy. Presentation of EMF can mimic other common conditions such as apical HCM, such as in our patient, constrictive pericarditis, rheumatic valve disease, or Ebstein’s anomaly making it difficult to diagnose. Furthermore, the rate of EMF progression can vary, and patients can present at any stage from the initial active phase with inflammation to a chronic phase typically free of inflammatory markers leading to restrictive cardiomyopathy [3].

Diagnosis of EMF is made primarily by echocardiography. Characteristic findings may include apical fibrosis of the ventricles, tethering of the atrioventricular valve papillary muscles, atrial enlargement, and restrictive filling pattern. A study in Mozambique found that biventricular EMF was the most common (55.5%) followed by right-sided EMF (28%). Left-sided EMF, such as in our patient, was found to be the least common at a prevalence of 16.6%. Furthermore, cMRI can visually demonstrate myocardial fibrosis in addition to myocardial edema, apical thrombus, and subendocardial delayed enhancement in the involved ventricles [2,7]. With cMRI, we were able to visualize an apical thrombus as well as underlying fibrosis thus helping us make the formal diagnosis of EMF in our patient. Though cMRI tends to be a difficult modality to obtain in the areas that are most affected, it can help differentiate EMF from other diseases as well as identify patients that may benefit from steroid therapy during the early stages of the disease [8].

Unfortunately, there is no effective medical therapy for EMF or intervention to prevent it. Potentially, it is theorized that patients with the presence of inflammatory markers, increased thrombolysis, and autoimmunity may benefit from the use of anti-inflammatory drugs, immunomodulators, and anticoagulants [2]. Mild to moderate EMF can be managed with the use of diuretics, renin-angiotensin system blockers, beta-blockers, anticoagulants, and corticosteroids mainly to improve symptoms of heart failure. Prognosis of EMF when untreated is very poor. Potential management options may be found with further research thus improving the mortality and morbidity of this rare disease.

## Conclusions

EMF is an extremely rare disease in the developed world that could be easily missed as a diagnosis. Though the diagnosis of EMF initially depends on echocardiographic findings, cMRI is an essential imaging modality that allows clinicians to easily differentiate between potential diagnoses with the information that it provides. Early diagnosis, differentiation, and treatment for HCM are important. Further research should provide more insight into the management of this rare disease.
